# Structure and Functions of Blood Vessels and Vascular Niches in Bone

**DOI:** 10.1155/2017/5046953

**Published:** 2017-09-17

**Authors:** Saravana K. Ramasamy

**Affiliations:** ^1^Institute of Clinical Sciences, Imperial College London, London W12 0NN, UK; ^2^MRC London Institute of Medical Sciences, Imperial College London, London W12 0NN, UK

## Abstract

Bone provides nurturing microenvironments for an array of cell types that coordinate important physiological functions of the skeleton, such as energy metabolism, mineral homeostasis, osteogenesis, and haematopoiesis. Endothelial cells form an intricate network of blood vessels that organises and sustains various microenvironments in bone. The recent identification of heterogeneity in the bone vasculature supports the existence of multiple vascular niches within the bone marrow compartment. A unique combination of cells and factors defining a particular microenvironment, supply regulatory signals to mediate a specific function. This review discusses recent developments in our understanding of vascular niches in bone that play a critical role in regulating the behaviour of multipotent haematopoietic and mesenchymal stem cells during development and homeostasis.

## 1. Introduction

Recent advancements in vascular biology have increased our understanding and knowledge of blood vessels and their characteristics during various physiological and pathological conditions. Blood vessels not only act as a transport conduit system but also play important roles in organ development, tissue morphogenesis, inflammation, barrier formation, and wound healing [1–4]. In addition, active involvement of blood vessels in the pathogenesis of a number of diseases suggests a fundamental need to understand these versatile transport networks in the body [5]. Blood vessels form an integral part of the skeletal system playing multiple roles in the maintenance of bone homeostasis. The importance of blood vessels in bone was initially recognised by surgeons during repair and healing of bone fractures [6, 7]. The essential role played by the bone vasculature during skeletal development [8–10] and fracture repair [6, 9, 11] has been an intense field of research. Further, the cell-specific contributions in pleiotropic functions of bone such as regulating whole body metabolism [12–14], brain functions [15–17], and mineral homeostasis [18–20] still need to be understood.

 Blood vessels in bone are reported to provide nurturing microenvironments to haematopoietic stem cells (HSCs) [21, 22] and mesenchymal stem cells (MSCs) [23, 24]. Various microenvironments in bone still need to be characterised well to understand their function during development, growth, and disease. Recent technical advances in bone imaging have substantially improved our fundamental knowledge of skeletal blood vessels. This review aims to provide an overview of recent developments and contemporary understandings of the bone vasculature and its microenvironments.

## 2. Structure and Characterisation of Blood Vessels in the Skeletal System

### 2.1. Skeletal Blood Circulation

**Figure 1:**
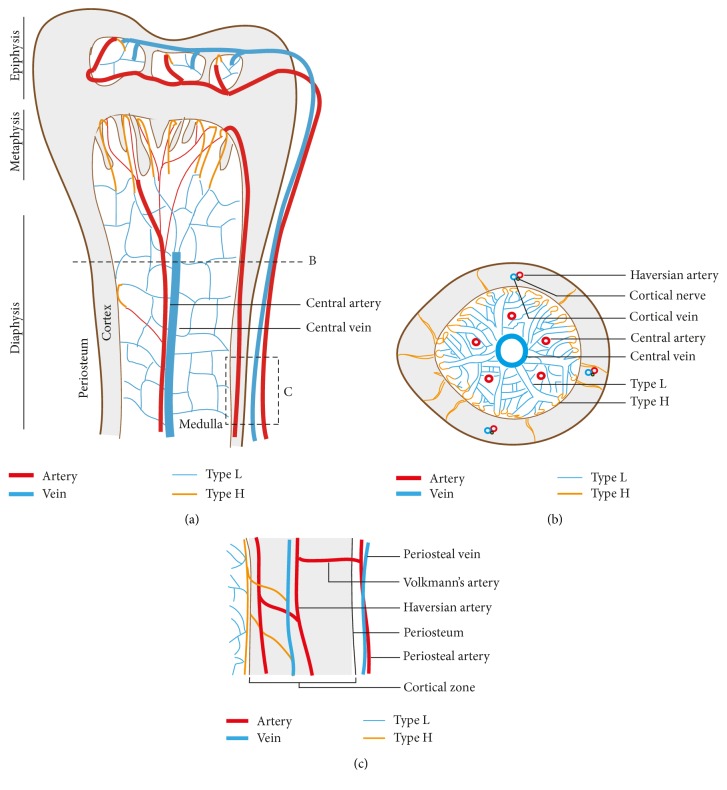
Blood vessel arrangement in long bone. (a) Longitudinal view demonstrates arrangement of arteries, veins, and capillaries in the epiphysis, metaphysis, and diaphysis regions of long bone. Arteries branch into smaller arterioles and terminate in type H capillaries. Type H capillaries are localised near osteoprogenitors in the metaphysis and endosteum regions. Type L capillaries are sinusoidal vessels terminating in the central vein. (b) Transverse view shows bone vascular pattern in cortical and medullary regions of long bone. A large central vein and a few nutrient arteries are prominent in the medullary region. (c) Arrangement of blood vessels showing the connection between cortical and medullary blood flow. Periosteal blood vessels are connected intermittently with cortical blood vessels.

Bone has an extensive network of blood vessels (Figure 1) consuming almost 10–15% of resting cardiac output [25, 26]. The spatial arrangement of blood vessels enables efficient and optimal delivery of oxygen and nutrients to various locations within the bone marrow compartment. Irrespective of the bone type, the main blood supply of bones is derived from arteries entering the cortical region, which connect with medullary sinusoids to finally exit the bone through veins [27, 28]. However, shape and type of skeleton can possibly affect the arrangement of capillary network existing between arteries and veins. Typical long bones, such as the femur and tibia, are supplied by several arteries and arterioles, which are classified based on their region of blood supply. The central artery also called as nutrient artery enters bone through a foramen and branches into a number of smaller arteries and arterioles to supply maximum regions of adult bone. It sustains high blood pressure to reach distant locations, usually terminating into capillaries present in the metaphysis and endosteum. There is a central large vein that receives blood from capillaries present in various regions and drains deoxygenated blood and nutrient waste from bone [29]. Periosteal arteries supply the outer surface of bone and are connected to Haversian arteries present in the cortical region through Volkmann's arteries. Haversian arteries run parallel to the longitudinal axis of the long bone in the cortex while shorter Volkmann's arteries run perpendicular to the long bone axis [30, 31]. Haversian arteries eventually converge into metaphyseal capillaries to deliver blood into the medullary region. In contrast, the blood supply from epiphyseal arteries does not have a route to enter the medullary region of long bones, thus maintaining a separate blood circulation in the epiphysis region. Epiphyseal arteries enter the bone from a heavy network of periarticular vascular plexus present near the ends of long bones. The veins draining the epiphyseal blood are relatively smaller compared to the vein present in the medullary region (Figure 1).

### 2.2. Heterogeneity in Blood Vessels

Divergence in arterial blood supply envisages the existence of multiple veins and capillary subtypes in bone. However, diversity within these blood vessels has not been well appreciated until recently. Fenestrated or sinusoidal capillaries form the majority of blood vessels in the skeletal vasculature. These are highly branched networks of blood vessels present in the marrow cavity of bones. Sinusoidal endothelial cells express vascular endothelial growth factor receptor-3 (VEGFR3) while bone arterial endothelium is negative for Vegfr3 [32]. Vascular structures in bone can be demarcated as laminin^+/low^Sca-1^−/low^ sinusoids, Sca-1^+^laminin^+^ endosteal vessels, and Sca-1^+^laminin^+^ central arteries [33]. Investigating blood vessels during postnatal development led to the identification of a new blood vessel subtype called type H present in actively growing regions of bone. They are named type H as they express high levels of blood vessel markers, endomucin (Emcn) and CD31 (Pecam1) compared to sinusoidal vessels, which express low levels of these markers thereby termed as type L [8, 10].

In an actively growing bone, type H vessels are present in the metaphysis and endosteum regions, while type L vessels predominate the whole medullary region. Type H capillaries are linearly structured, columnarly arranged blood vessels in comparison to a branched network of type L capillaries. The leading fronts of type H vessels, which mediate angiogenesis in bone, contain bulge-shaped lumenised structures [10, 29]. However, the functional significance of these unique structures in the vascular front remains unknown. Arteries and arterioles express ephrin B2 (Efnb2) and are negative for Emcn expression. A subpopulation of endothelial cells within type H endothelium, expressing both Efnb2 and Emcn, is proposed to generate arteriolar blood vessels (Efnb2+, Emcn−). This subfraction of type H blood vessels displays expression of other arterial markers such as Sox17 and neuropilin-1 [34]. Arteries are tightly enwrapped by *α*-smooth muscle actin+ (*α*SMA+) mesenchymal cells, while smaller arterioles have *α*SMA− and platelet-derived growth factor receptor beta+ (PDGFR*β*+) perivascular cells. Multiple types of bone mesenchymal cells and their association with blood vessel subtypes are discussed later in this review. Thus, the bone vasculature is heterogeneous, unique, and needs profound investigation to understand tissue-specific vascular modifications and specialised functions.

## 3. Blood Flow and Oxygenation in Bone

The spatial arrangement of blood vessels is intricate and unique in every tissue to provide proper oxygen and nutrient supply to the whole tissue or organ. The organisation of distinct blood vessel subtypes in long bones indicates a peculiar blood flow pattern. Blood velocity is higher in type H vessels compared to type L vessels. When blood flows down from type H capillaries, blood velocity drops with each vascular branch in the metaphysis to attain a characteristic low velocity for type L capillaries in the diaphysis. Frequent branching and joining of vascular networks in the diaphysis maintain low blood velocity in the diaphyseal capillaries [29, 35].

### 3.1. Oxygen Status in Bone Vascular Microenvironments

The peculiar blood flow pattern in bone coincides with oxygen status of the bone microenvironment. Measurement of local oxygen tension (pO_2_) in live mice indicated that pO_2_ is higher in the endosteal bone region than in the deeper sinusoidal regions. Endosteal regions are vascularised by type H capillaries and arterioles compared to type L vessels in sinusoidal regions [36]. It has also been illustrated that low vascular permeability in arterial and type H vessels maintain low reactive oxygen species (ROS) in the microenvironment compared to fenestrated, highly permeable sinusoids [35]. Analysing HSCs in Hoechst-perfused mice showed that the localisation of long-term HSCs (LT-HSCs) is limited to the least perfused regions in the BM [37, 38]. The low oxygen or hypoxic microenvironment supports maintenance of HSCs and protects them from damage caused by oxygen stress [39]. Hypoxia-dependent stabilisation of hypoxia inducible factor (HIF) is essential for the canonical HIF-mediated signalling pathway that plays divergent roles in blood vessels [8, 34], mesenchymal cells [40], and haematopoietic cells [39, 41, 42] in the BM microenvironment.

Hypoxia and HIF-1*α*-mediated regulation of chondrocyte growth and survival is essential for chondrogenesis and growth plate development [43, 44]. An important downstream target of HIF-1*α* is VEGF, a fundamental factor required for blood vessel formation in physiological and pathological conditions [45]. VEGF plays a pleiotropic role in regulating several processes during bone development, growth, and repair [46]. Genetic studies in chondrocytes illustrated essential functions of their VEGF in angiogenesis and bone formation in addition to regulating chondrogenesis [47, 48]. Thus, hypoxia-mediated regulation of HIF controls VEGF levels to couple blood vessel growth and osteogenesis in bone [49, 50]. Stabilisation of HIF in osteoprogenitors results in the expansion of the HSC niche and promotion of erythropoietin production in bone [51]. HSCs also exploit HIF signalling to precisely regulate their cell cycle and quiescence status in the BM [42].

Genetic and pharmacological manipulations of blood flow in developing zebra fish affected nitric oxide synthase signalling in primitive HSCs resulting in defective HSC development [52]. Blood flow is also an important player in mobilising haematopoietic cells from bones to various organs and tissues. In mice, sinusoidal vessels having high permeability promote migration and differentiation of HSPCs [35]. A declining number of type H vessels and arterioles with age in bone leads to reduced skeletal blood perfusion and HSC function [29, 34]. In addition, manipulating blood flow in bone leads to defective angiogenesis and bone formation [29], suggesting blood flow as a potential cause of age-related bone loss. These compelling evidences argue the importance of blood flow in maintaining skeletal homeostasis by regulating bone formation and haematopoiesis.

### 3.2. Clinical Importance of Blood Flow in the Skeleton

Despite differences in bone structures, studies from rodent models have been beneficial for the development of therapeutic strategies to target human bone diseases. Basic understanding of blood vessels and blood flow in the skeletal system is mainly derived from findings in rodent models. Rodents show age-related bone loss similar to humans. Remarkably, identification of decrease in bone arterial capillaries with age in mice [34] corresponds with age-associated decline in femoral arterial blood flow in humans [53]. Recent demonstration of decreased type H vessels in aged and osteoporotic human subjects [54] highlights the significance of investigating skeletal blood vessels in rodents.

Increasing number of clinical evidences indicate the importance of blood flow in maintaining homeostasis of the skeletal system. Reduced blood supply was measured in bones of elderly women with osteoporotic conditions [55]. Impairment of blood supply to bone causes death of bone cells leading to the development of osteonecrosis condition [56]. A comparative study in patients with unilateral arterial occlusive disease showed deleterious effect of defective blood flow on bone mineralisation [57]. Defects in blood flow in the subchondral region has been identified as a potential mechanism in generating osteoarthritis [58]. Systemic disorders such as diabetes [59], chronic obstructive pulmonary disorders [60], and hypertension [61] that impair vascular perfusion are associated with bone defects. Moreover, blood supply is critical for initiation of callus formation during fracture healing and repair [6]. Defective blood vessel formation is observed at fracture sites showing delayed healing and regeneration processes [50]. Disuse-induced osteopenia conditions such as bed rest and hindlimb unloading have also been associated with changes in blood supply to bone [62, 63]. In spite of clinical data supporting experimental findings, further research is required to understand molecular mechanisms involved in the generation of these clinical conditions.

## 4. The Vascular Microenvironment for Mesenchymal Cells

### 4.1. Types of Mesenchymal Stem and Progenitor Cells Forming Bone Marrow Stroma

Mesenchymal lineage cells comprising a majority of bone marrow stromal cell population form an important component of the bone marrow microenvironment. Multipotent mesenchymal stem and progenitor cells (MSPCs) can generate various types of bone marrow mesenchymal stromal cells including osteoblasts, chondrocytes, adipocytes, and reticular cells. Understanding the hierarchical relationship of BM stromal cells is still an intensive area of research. Although perivascular origin of MSPCs in different organs has been suggested [23], distinct waves of stromal cells have been identified in the developing bone marrow [64, 65]. Genetic lineage tracing techniques has provided significant knowledge in understanding the heterogeneity associated with BM mesenchymal cells. Nestin-GFP+ cells wrap endothelial cells (ECs) that form arteries and type H capillaries. Perivascular Nestin-GFP+ cells were identified to mark early MSPCs, which can generate bone marrow stroma and bone-lineage cells [66]. Similarly, osterix+ neonatal mesenchymal cells possess the potential to generate bone lineage cells, chondrocytes, adipocytes, and BM stroma. In contrast, osterix+ embryonic and adult mesenchymal cells show limited potential [64]. Remarkably, both osterix+ and Nestin-GFP+ cells are present near type H capillaries and absent around perisinusoidal type L capillaries [8, 35]. Perisinusoidal stromal cells expressing leptin receptor (LepR) were suggested to contribute to bone-lineage cells when marked early during development [67]. Remarkably, LepR expression in adult mesenchymal cells promotes their adipogenic potential inhibiting osteogenic cell fate [68]. LepR+ cells contribute to C-X-C motif chemokine ligand 12 (Cxcl12) expressing cells in the bone marrow [69]. Cxcl12 expressing Nestin-negative mesenchymal cells provide the HSC supporting microenvironment [70]. Therefore, it will be interesting to use an inducible (–*CreER*) system to understand stage-specific contribution of LepR+ cells in BM stroma.

In contrary to perivascular MSPCs, cells of nonperivascular origin have also been identified to contribute to bone lineage cells and BM stroma. Lineage tracing cells of chondrogenic origin using *Col2-CreER* system demonstrated their potential to form bone lineage cells and Cxcl12-abundant reticular stromal cells [65]. Similarly, lineage tracing using other chondrogenic systems such as Sox9- and Aggrecan-*CreER* also confirmed the cells' potential to generate multiple mesenchymal lineage cells. Identification of Gremlin1 as a marker for cells with osteochondroreticular potential indicates the possible existence of distinct progenitor subtypes within the pool of MSPCs. Clonally expanding Gremlin1+ cells were identified in growth plate and metaphysis region and they lack adipocyte differentiation potential [71]. These studies demonstrate the existence of heterogeneity in MSPCs and need to understand subtypes within the population to identify their hierarchical relationship.

### 4.2. Localisation of Mesenchymal Stromal Cells in the Vascular Niche

**Figure 2:**
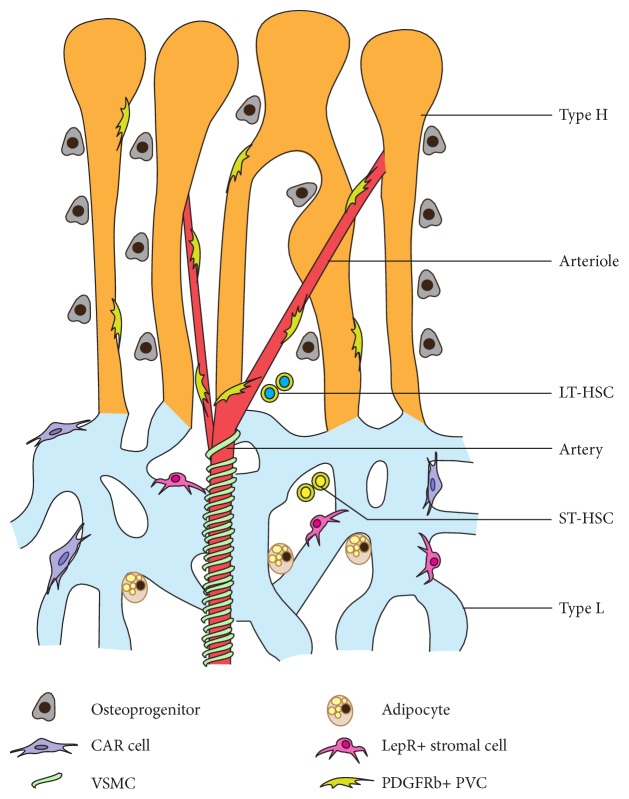
Vascular microenvironments in bone. Multiple types of perivascular mesenchymal stromal cells are supported by distinct subtypes of vascular structures in the bone marrow microenvironment. Arteriolar niche supports long-term HSCs (LT-HSC) while sinusoidal niche maintains short-term and cycling HSCs (ST-HSCs).

Localisation of MSPCs suggests that multiple regions within the bone marrow microenvironment can support and provide niches for MSPCs. Col2+, Sox9+, and Aggrecan+ cells are located on the growth plate, which is an avascular region [65]. Gremlin1+ cells are present in both growth plate and metaphysis regions [71]. Nestin-GFP+ cells are located around arteries and in the metaphysis [35, 66]. PDGFR*β*+ mesenchymal cells show a distribution pattern similar to Nestin-GFP+ cells [34]. Majority of osterix+ cells are located around type H vessels in the metaphysis [8, 64]. LepR+ and Cxcl12+ cells are largely localised around type L (perisinusoidal) vessels [67, 68]. Chondrocytes are present in the avascular zone, typically in the growth plate or epiphysis region of bones [65]. Osteogenic progenitors are specifically localised around type H vessels in the metaphysis and endosteum regions [8]. Fat cells or adipocytes preferentially present in perisinusoidal space of the diaphysis [68]. Reticular cells are also localised around type L vessels in the perisinusoidal region [67, 69]. Vascular smooth muscle cells are *α*SMA+ periarterial cells, tightly wrapping arteries in the bone marrow microenvironment [8, 34]. Thus, subpopulations of heterogenic BM mesenchymal stromal cells preferentially localise around specific blood vessel subtypes, suggesting the existence of specialised vascular microenvironments (Figure 2()).

Evidences suggest the central role played by blood vessels in supporting the local microenvironment. High expression of pro-osteogenic factors in type H vessels generates the microenvironment required for osteoprogenitors. Promoting type H capillaries in bone results in improved osteoprogenitor numbers [8, 10]. Similarly, platelet-derived growth factor B (PDGF-B) released by endothelium binds to PDGF receptor present on mesenchymal cells to activate growth mediated signalling pathways [72]. Overexpression of PDGF-B in bone endothelium results in increased PDGFR*β*+ perivascular mesenchymal cells in the bone marrow [34]. Mesenchymal cells also release angiogenic factors such as VEGF, angiopoietin, FGF, and BMP [3, 4] to maintain a mutual relationship in regulating a specific bone marrow microenvironment.

## 5. Blood Vessels in the Haematopoietic Stem Cell (HSC) Microenvironment

### 5.1. Bone Endothelial Cells in HSC Maintenance

A strong interdependence of ECs and HSCs has been illustrated during both primitive and definite haematopoiesis [22, 73, 74]. The importance of the BM vasculature was initially appreciated only in thrombopoiesis, stem cell mobilization, and homing [21]. Identification of long-term (LT) HSCs' occurrence near blood vessels generated an immense interest in the field to understand the bone marrow vascular niche [75]. Cultured ECs from nonhaematopoietic organs such as heart and liver were identified to maintain HSCs *in vitro*, while ECs from kidney lacked this potential [76]. Later, identification of tissue-specific molecular signals in ECs [77] suggested unique potential of bone marrow endothelium in profoundly supporting HSCs and haematopoiesis compared to ECs from other organs.

Endothelial specific deletion of glycoprotein 130 (gp130), a subunit of receptors that bind IL-6 chemokine family, resulted in hypocellular marrow and reduced HSC numbers [78]. Regeneration of sinusoidal ECs after irradiation was severely affected upon inhibiting VEGFR2 signalling with a blocking antibody [79]. E-selectin was suggested to be exclusively expressed in the bone marrow endothelium, and deletion of this gene enhances HSC quiescence and resistance to irradiation [80]. In addition to direct cell contact, ECs were illustrated to release soluble factors called angiocrine factors to regulate the HSC microenvironment [2]. Cxcl12 and stem cell factor (Scf) are important and widely investigated angiocrine factors of BM ECs involved in regulating HSC homeostasis. Endothelial cell-specific deletion of Scf resulted in decreased HSC numbers with reduced repopulation potential upon BM transplantation [81]. In a similar study, deletion of Cxcl12 in ECs resulted in depletion of HSCs and their long-term repopulating activity [69]. In a recent study, activation of Notch signalling in ECs led to the expansion of both cellular and angiocrine components of the HSC microenvironment. Endothelial Notch signalling promoted formation of new type H capillaries, small arterioles, PDGFR*β*+ perivascular mesenchymal cells, and cellular Scf levels [34].

### 5.2. Arteriolar Microenvironments for Long-Term HSCs

Arteriolar microenvironments consisting of arterial ECs and surrounding NG2+ mesenchymal cells were demonstrated to maintain HSC in a quiescent state [82]. Similarly, sinusoidal blood vessels and surrounding LepR+ mesenchymal cells were also shown to provide microenvironments to maintain HSC population [83]. In another study, *α*-catulin GFP+ c-Kit+ HSCs were shown to localise in the central marrow region consisting of a sinusoidal microenvironment formed by sinusoidal blood vessels and LepR+ mesenchymal cells. It also proposes a single perisinusoidal microenvironment for both quiescent and dividing HSCs [84]. Arterial blood vessels having less permeability were shown to maintain HSC in a low ROS compared to highly permeable sinusoids involved in cell trafficking and homing [34, 35]. Endothelial Notch signalling-mediated amplification of arteriolar blood vessels leads to the expansion of HSC niches that result in increased HSC numbers and their function in young mice [34]. Recently, Hoxb5 expression in BM was identified to demarcate LT-HSCs population. Spatial localisation of Hoxb5+ HSCs shows that they are directly attached to VE-cadherin+ ECs, indicating their close association with blood vessels in the BM microenvironment [85]. The study does not provide further details regarding the vascular microenvironment near Hoxb5+ HSCs. It will be interesting to understand the localisation of Hoxb5+ HSCs in the context of multiple vascular compartments present in the BM microenvironment.

## 6. Concluding Remarks

Despite emerging interest in the bone vasculature and that manipulating blood vessels might regulate the BM microenvironment, our knowledge of heterogeneous vascular niches and endothelial regulatory factors is limited, to gain insight into the vessel-mediated organisation of the BM microenvironment.  Table 1 summarises important factors studied in bone endothelial cells and their specific functions. It has become increasingly evident that the bone vasculature is highly complex, heterogeneously composed of distinct blood vessel types, and endowed with specialized functions that control bone formation, haematopoiesis, and bone regeneration. ECs forming these heterogeneous blood vessels along with their released angiocrine factors and supporting surrounding cell types contribute to the formation of multiple microenvironments in the bone marrow. In addition, local oxygen status generated by the organisation of capillaries and microcirculation regulates the behaviour and functions of microenvironments. Involvement of multiple factors and cell types suggests the existence of disciplined regulation mechanisms to control the integrity of local niches. Dissecting cellular and molecular components of these local microenvironments will enhance our understanding of clinically significant HSCs and MSCs in bone.

Bone mass is severely affected in physiological changes such as ageing and in systemic diseases such as diabetes [86] and hypothyroidism [87]. The BM microenvironment is modified in accordance with these physiological and pathological conditions in the body. These changes perhaps involve amplification or reduction of a specific microenvironment within the bone marrow compartment to compensate changes in the whole body physiology. For example, age-related physiological changes lead to loss of bone mass and are associated with the loss of type H vessels which provide the supportive microenvironment for osteoprogenitors [8]. Similarly, changes in the BM microenvironment were observed during cancer and metastasis [88, 89]. These evidences strongly argue that the dynamic nature of the bone marrow microenvironment undergoes modifications based on the local and systemic demands and functions.

ECs playing a central role in constructing and orchestrating various microenvironments in the BM could potentially serve as an excellent target to manipulate specific niches in bone. Reactivation of type H vessels in aged mice could promote neo-osteogenesis, leading to new bone formation and increase in bone mass [8, 29]. Despite its potential therapeutic applications, limited knowledge of the bone vasculature severely affects our understanding of the organisation and localisation of microenvironments in bone. Characterising heterogeneous blood vessels and their endothelial-derived factors and further insights on the cellular and molecular components of microenvironments are critical to unravel the interaction and role of blood vessels in regulating the bone marrow architecture in various physiological and pathological conditions.

## Figures and Tables

**Table 1:** Genetic studies illustrating functions of endothelial factors in bone are summarised below.

Factors	Modification	Functions	Reference(s)
Cxcr4	EC-specific deletion (induced)	Increased vascular permeability HSPC egress	[35]
Cxcl12	EC-specific deletion (constitutive)	Decreased HSC frequency	[69, 70]
Dll1	EC-specific deletion (induced)	Monocyte development	[90]
Dll4	EC-specific deletion (induced)	Regulates type H vessels Coupling of angiogenesis and osteogenesis haematopoiesis	[10, 34]
Fbw7	EC-specific deletion (induced)	Reactivating type H vessels in aged bones induce arterioles formation increase PDGFRb+, alpha-SMA+ mesenchymal cells increase HSC frequency	[10, 29, 34]
Fgfr1/2	EC-specific deletion (induced)	Impaired vascular integrity reduced HSPCs and MSPCs	[35]
Gp130	EC-specific deletion (constitutive)	Hypocellular marrow, marrow dysfunction, and splenomegaly	[78]
Hif1a Vhl	EC-specific deletion (induced)	Regulates type H vessels Coupling of angiogenesis and osteogenesis	[8]
Pdgfb	EC-specific overexpression (induced)	Increased PDGFRb+, alpha-SMA+ mesenchymal cells	[34]
Pecam1	Global deletion	No substantial change in blood vessels	[29]
Scf	EC-specific deletion (constitutive)	Decreased HSC frequency	[81]
Sele	Global deletion	Promotes HSC quiescence and resistant to irradiation	[80]
